# The Use of Dornase Alfa in the Management of COVID-19-Associated Adult Respiratory Distress Syndrome

**DOI:** 10.1155/2021/8881115

**Published:** 2021-04-23

**Authors:** Andrew Toma, Christina Darwish, Michele Taylor, Justin Harlacher, Ribal Darwish

**Affiliations:** ^1^Palm Beach Gardens Medical Center, Palm Beach Gardens, FL, USA; ^2^George Washington University, School of Medicine and Health Sciences, Washington, DC, USA

## Abstract

**Objective:**

Currently, management of acute respiratory distress syndrome (ARDS) in COVID-19 infection with invasive mechanical ventilation results in poor prognosis and high mortality rates. Interventions to reduce ventilatory requirements or preclude their needs should be evaluated in order to improve survival rates in critically ill patients. Formation of neutrophil extracellular traps (NETs) during the innate immune response could be a contributing factor to the pulmonary pathology. This study suggests the use of dornase alfa, a recombinant DNAse I that lyses NETs, to reduce ventilatory requirements and improve oxygenation status, as well as outcomes in critically ill patients with ARDS subsequent to confirmed or highly suspected COVID-19 infection.

**Design:**

A single-institution cohort study. *Setting*. Intensive care unit in a tertiary medical center. *Patients*. Adult patients with acute respiratory distress syndrome (ARDS) admitted to the ICU with confirmed COVID-19 infection. *Intervention*. Treatment with aerosolized dornase alfa. *Measurements and Main Results*. Of 39 patients evaluated, most patients had improvement in oxygenation measured by increase in the PaO_2_/FiO_2_ ratio, reduction in ventilatory support or other supportive oxygen requirements, and partial resolution of bilateral opacities visible on CXR, as well as improved outcome.

**Conclusions:**

Administration of inhalational dornase alfa via a filtered nebulizer medication system or through an adapter in a ventilator circuit should be considered in all COVID-19-positive patients with ARDS as early in the disease course as possible.

## 1. Introduction

As of May 31, 2020, the SARS-CoV-2 (COVID-19) pandemic has resulted in more than 6 million confirmed cases worldwide and over 369,000 deaths [1]. Among critically ill patients, hypoxemic respiratory failure from acute respiratory distress syndrome (ARDS) is the predominant finding and leading cause of death [2]. Currently, debate surrounds the pathophysiology of ARDS in these patients and how to standardize appropriate treatment [3]. Patients requiring invasive mechanical ventilation have a dismal prognosis, with one study of 2634 patients in the New York City area determining mortality rates ranging from 76.4% to 97.2%, depending on age [4]. It is therefore imperative to identify possible treatments that will preclude ventilator requirement or reduce necessary duration of its use.

One proposed contributor of pulmonary disease in COVID-19 is the presence of neutrophil extracellular traps (NETs) resulting from the initial innate immune response [5, 6]. These are web-like structures composed of chromatin which are responsible for entrapping pathogens and further propagating the immune response. With dysregulation, significant pulmonary pathology can occur from alveolar damage and epithelial cell death [7]. NETs may also contribute to the formation of viscous mucous, resulting in impaired gas exchange and facilitation of secondary infections [6]. In an apparent paradox to their role as effectors of pathogen clearance, a study of pneumonia correlated a higher concentration of NETs with prolonged treatment duration and inpatient stay [8]. NETs have also been implicated in pathogenesis of ARDS, as toxic extracellular histones are elevated in the bronchoalveolar fluid of these patients [6]. Finally, NETs may potentiate thrombosis by activating components of the clotting cascade, inactivating endogenous anticoagulants, and supporting recruitment of platelets [9]. This is especially concerning because COVID-19 has been associated with thrombotic complications including pulmonary embolism in critically ill patients [10].

Dissolution of NETs is possible with dornase alfa, a recombinant DNAse I used primarily in the treatment of cystic fibrosis, that has previously demonstrated success in ARDS treatment [6, 11, 12]. A recent case report showed promise in its use for a patient with COVID-19, who had improved oxygenation and lung compliance with eventual extubation and discharge [5]. In this study, we report a cohort study that examined the possible role of dornase alfa as a therapeutic modality in ARDS resulting from COVID-19 infection. The goal was to observe whether this treatment led to a decrease in ventilatory requirements for patients requiring mechanical ventilation, an improvement in oxygenation status, and a resolution of pulmonary infiltrates radiographically.

## 2. Methods

This is a retrospective single-institution cohort study conducted from April 4, 2020, to July 4, 2020, in the intensive care unit at Palm Beach Gardens Medical Center. Approval for this study was obtained from MetroWest Medical Center Institutional Review Board (IRB# 2020-063). Patients were considered eligible if they had positive COVID-19 infection confirmed by nasopharyngeal RT-PCR and were diagnosed with ARDS. Diagnosis of ARDS required fulfilment of Berlin criteria including timing within 1 week of clinical insult or new/worsening respiratory symptoms, chest X-ray (CXR) showing bilateral opacities not fully explained by effusions, lobar/lung collapse, or nodules, and respiratory failure not fully explained by cardiac/fluid overload. Severity grading was based on the PaO_2_/FiO_2_ ratio: mild PaO_2_/FiO_2_ >200 to ≤300 mmHg with PEEP or CPAP ≥5 cmH_2_O, moderate PaO_2_/FiO_2_ >100 to ≤200 mmHg with PEEP or CPAP ≥5 cmH_2_O, and severe with PaO_2_/FiO_2_ <100 mmHg with PEEP ≥5 cmH_2_O. The exclusion criteria were known allergy to doranase alfa, DNR status prior to initiation of treatment, and hemoptysis on presentation.

**Figure 1:**
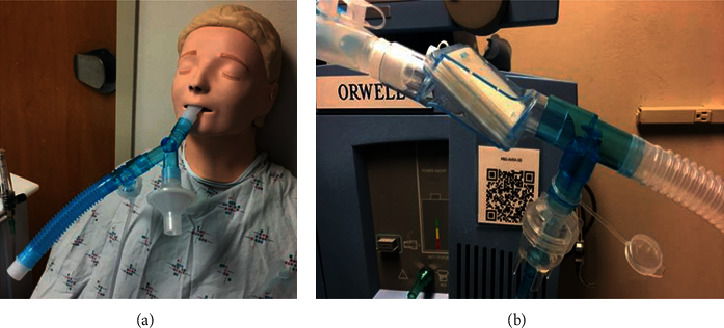
Demonstration of mechanism utilized for aerosolized dornase alfa administration: (a) AirLife® Misty Max 10^TM^ nebulizer medication system with filter and (b) AirLife® Tee adapter with valve in conjunction with a small volume nebulizer.

Eligible patients not requiring invasive ventilation received 2.5 mg aerosolized dornase alfa daily or twice daily via a nebulizer medication system with filter. This mechanism utilizes a reservoir and one-way valve system for medication administration to protect clinicians against secondhand aerosols (Figure 1(a)). Eligible patients requiring mechanical ventilation received 2.5 mg aerosolized dornase alfa daily or twice daily via a tee adapter with valve in conjunction with a small volume nebulizer. The tee adapter introduces the aerosol into the ventilator circuit and maintains a closed system to reduce the risk of aerosolization (Figure 1(b)). To further reduce exposure risk, for patients not admitted to negative pressure rooms, clinical staff were not permitted to enter for an hour after the nebulizer treatment.

Outcomes of interest included changes in ventilatory requirements, oxygenation measured by comparing the arterial oxygen partial pressure (PaO_2_) over the fractional inspired oxygen (FiO_2_), and appearance of infiltrates on CXR before and after treatment, as well as outcome at 3 months after treatment.

## 3. Results

Of 39 patients included in the study, 25 (62.5%) were males, and the mean age was 63.5 years (median = 64 years). The majority (*n* = 37) had COVID-19 infection confirmed by RT-PCR, and the remaining had positive IgM titers (*n* = 2). The number of treatments of dornase alfa ranged from 3 to 43 with an average of 9.05. Prior to treatment with dornase alfa, 8 patients (20%) required intubation and mechanical ventilation, 10 required BiPap (25%), 9 required high-flow nasal cannula (23%), 11 required midflow nasal cannula (28%), and 1 (2.5%) was stable on face mask. Within the first week after initial treatment, the majority of patients (*n* = 24, 61.5%) had reduced respiratory support requirements (e.g., transition from BiPap to nasal cannula). By the end of the study period, of 8 patients initially requiring mechanical ventilation, 7 were successfully extubated or weaned to tracheal collar and discharged from the ICU (Table 1). In a sample of the first 10 patients included in the study, all had improved oxygenation measured by an increase in the PaO_2_/FiO_2_ ratio after treatment, with an average increase in the ratio by 146.02% (median: 144.5%, 37.33%–322.67%). The average improvement was higher among patients not initially requiring mechanical ventilation (184.55%) compared to those requiring it (120.34%) (Table 2).

**Figure 2:**
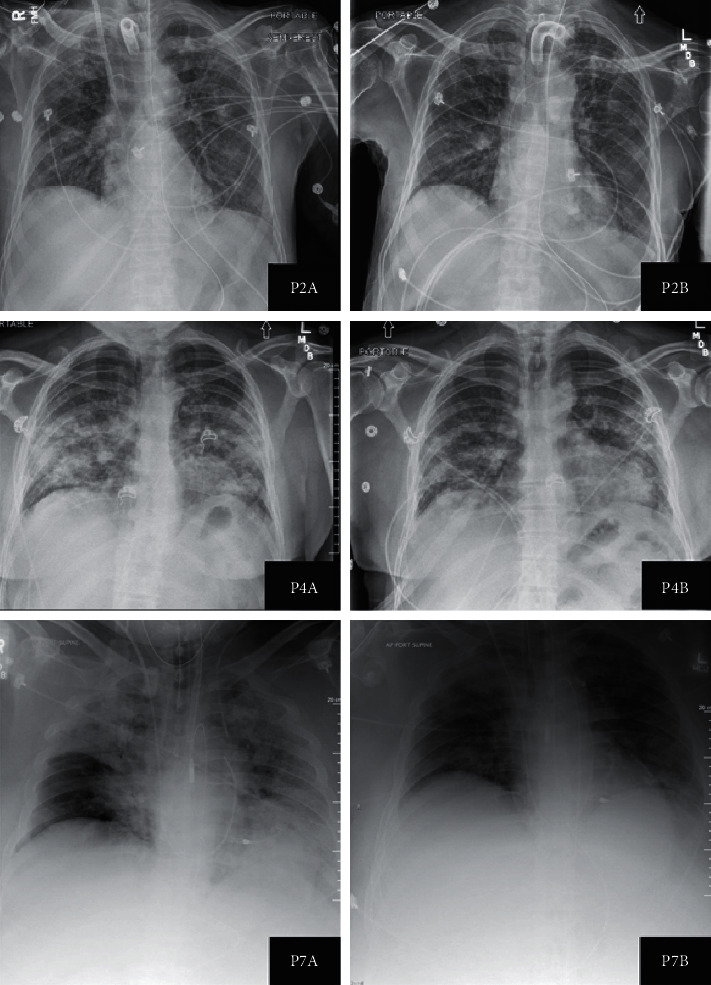
Chest X-rays of patients II, IV, and VII before (A) and after (B) treatment with dornase alfa. Imaging demonstrates improved resolution of opacities including for those who had prolonged mechanical ventilation (patients II, P2A/B, and VII, P7A/B) and for those not requiring mechanical ventilation (patient IV, P4A/B).

All patients also had improvement in bilateral opacities visible on CXR, and this is demonstrated for patients II, IV, and VII in (Figure 2).

## 4. Discussion

Though inhalational dornase alfa is most commonly utilized in the prevention and treatment of pulmonary complications in cystic fibrosis, our study results suggest a potential benefit to its use in ARDS caused by COVID-19. The role of NETs in pulmonary disease is well documented, and the ability of dornase alfa to mitigate the activity of this component of the innate immune response likely reduces the deleterious secondary inflammation that results from COVID-19. We developed a unique strategy to ensure safe administration of the drug to both ventilated and nonventilated patients in order to reduce aerosolization of the virus and exposure to health care workers. Our cohort study anticipates a randomized clinical trial currently underway that likewise is assessing the benefit of dornase alfa in COVID-19 disease but is not scheduled to be completed until November 2020. Since the reported adverse events of treatment, including rash and voice changes, are rare and relatively mild, we offer use of inhaled dornase alfa as a supplement to current supportive measures used to treat ARDS in select COVID-19 patients until confirmatory data are published [5].

In our study cohort, most of the patients who received doranase alfa had improvement in oxygenation and radiogragraphic resolution of the pulmonary infiltrates. Most of the patients who required mechanical ventilation had reduced ventilatory requirements or were successfully extubated by the end of the duration of the study. The majority of patients who were treated prior to need for mechanical ventilation were able to be discharged from the ICU, and none progressed to requiring mechanical ventilation. Of note, patients who had been intubated for a prolonged period prior to receiving treatment with dornase alfa had more modest improvements in respiratory status. This finding, compared to the more dramatic results in those who did not yet require invasive mechanical ventilation, suggests dornase alfa has higher efficacy when used earlier in the disease course. It is likely that once significant inflammation has resulted from the NETs, gas exchange could already be permanently impaired. In a patient in whom ARDS was secondary to COVID-19 and pesticide exposure, doranase alfa showed no efficacy.

Limitations of our study include retrospective design with a small control group (Table 3), small sample size, limited follow-up to three months, and younger average age group compared to other studies [4]. Given the small sample size, it was also not possible to adjust for other factors that may have impacted the patient's success with treatment, including length of hospitalization, ventilation prior to initiation of dornase alfa, or underlying comorbidities.

## 5. Conclusion

We present a cohort study showing efficacy and safety of a novel administration method of inhalational dornase alfa to both spontaneously breathing and mechanically ventilated patients with ARDS due to COVID-19 infection using a one-way valve and viral filter. Our data show improved outcome and safety of utilization of doranase alfa in the treatment of ARDS. Future randomized controlled trials will be needed to confirm the generalizability of these results to the broader population of COVID-19-infected patients.

## Figures and Tables

**Table 1:** Characteristics of the patients, severity of the ARDS, number of doranase alfa treatments, and outcome in the treatment group.

Characteristics	Number of patients (*N* = 39)	Outcome	Home	LTAC	Death/hospice
Average age	64		33	2	4
Male	25		22	1	2
Female	14		11	1	2

*Number of dornase alfa treatments*					
1–4	8		7	0	1
5–10	23		22	0	1
11–15	3		2	0	1
>15	5		2	2	1

*ARDS severity*					
Mild	22		22	0	0
Moderate	7		7	0	0
Severe	10		4	2	4

*Comorbidities*					
HTN	16		15	0	1
HLD	5		4	0	1
DM	6		4	2	0
Malignancy	2		1	1	0
Transplant recipient	1		0	1	0
MI/CMO	3		3	0	0
PAF	3		2	1	0
Obstructive or restrictive lung disease	5		3	0	2
Pesticide exposure	1		0	0	1
CKD	3		2	1	0

*Additional treatments*					
Dexamethasone	20		20	0	0
Dexamethasone + remdesivir	15		12	0	3
Dexamethasone + remdesivir + plasma	1		0	1	0
Dexamethasone + plasma + remdesivir + hemolung	3		1	1	1

**Table 2:** Characteristics of the first 10 patients, including changes in the ventilator requirements and PaO_2_/FiO_2_ ratio (ventilatory and clinical features of critically ill patients with COVID-19 infection and ARDS treated with dornase alfa).

	Patient I	Patient II	Patient III	Patient IV	Patient V	Patient VI	Patient VII	Patient VIII	Patient IX	Patient X
Age	72	68	48	62	68	42	48	55	79	35

Sex	M	M	F	M	F	M	M	M	M	M
Comorbidities	Renal transplant, type II DM, prior history of malignancy	—	PAF, type II DM	—	HTN	—	CAD with prior STEMI	—	Pulmonary fibrosis	CM

COVID+ by RT-PCR	Yes	Yes	Yes	Yes	Yes	Yes	No	No	Yes	No
Remdesivir use	Yes	Yes	Yes	No	No	No	No	No	No	No

Respiratory support (before) Pressure (cmH_2_O) Volume (mL)	PCV: 28/12	PRVC TV: 450 mL VR: 32 bpm PEEP: 14	PRVC TV: 450 mL VR: 30 bpm PEEP: 16	HFNC	HFNC	BiPap	PRVC TV: 450 mL VR: 22 bpm PEEP: 12	BiPap	PCV 22/12	PRVC TV: 450 mL VR: 22 bpm PEEP: 10

Respiratory support (after) Pressure (cmH_2_O) Volume (mL)	SIMV TV: 500 mL Rate: 10 bpm PEEP: 5 PS: 10	SIMV TV: 500 mL Rate: 15 bpm PEEP: 5 PS: 16	PRVC TV: 480 mL Rate: 30 bpm PEEP: 10	NC	NC	NC	NC	NC	PCV: 16/8	NC

PaO_2_ (mmHg)/FiO_2_ (%) before	138 (69/50)	240 (120/50)	84 (100/84)	76 (61/80)	150 (60/40)	208 (104/50)	89 (80/90)	75 (100/75)	148 (89/60)	90 (90/100)

PaO_2_ (mmHg)/FiO_2_ (%) after	202 (101/50)	350 (140/40)	133 (80/60)	275 (88/32)	206 (66/32)	450 (144/32)	272 (87/32)	317 (114/36)	405 (243/60)	263 (84/32)

% increase in PaO_2_/FiO_2_	46.38	45.83	58.33	261.84	37.33	116.35	205.62	322.67	173.65	192.22
No. of treatments	9	16	14	4	4	3	4	4	2	7
Extubated	s/p trach	Yes	Yes	N/A	N/A	N/A	Yes	N/A	No	Yes

Discharged from ICU	Yes	Yes	Yes	Yes	Yes	Yes	Yes	Yes	No	Yes

Abbreviations: PAF: paroxysmal atrial fibrillation; DM: diabetes mellitus; CM: cardiomyopathy; CAD: coronary artery disease; STEMI: ST-elevation myocardial infarction; ICU: intensive care unit; PCV: pressure-controlled ventilation; PRVC: pressure-regulated volume control; HFNC: high-flow nasal cannula; NC: nasal cannula; SIMV: synchronized intermittent mandatory ventilation; TV: tidal volume; PEEP: positive end-expiratory pressure; PS: pressure support; VR: ventilatory rate; BPM: breaths per minute.

**Table 3:** Characteristics of patients with ARDS and outcome of patients who were not treated with doranase alfa.

Characteristics	Number of patients (*N* = 6)	Outcome	Home	LTAC	Death/hospice
Average age	65		1	1	4
Male	4		0	1	3
Female	2		1	0	1

*Comorbidities*					
Hypothyroidism	1		1	0	0
CKD	3		0	1	2
HIV	1		0	0	1
CAD	1		0	0	1

*Treatments*					
Dexamethasone	3		1	1	1
Solu-Medrol IV	1		0	0	1
Dexamethasone + remdesivir	0		0	0	0
Dexamethasone + plasma	1		0	0	1
Dexamethasone + plasma + hemolung	1		0	0	1

## Data Availability

The data used to support this study are available from the corresponding author upon request.
